# Aberrant hypomethylation at imprinted differentially methylated regions is involved in biparental placental mesenchymal dysplasia

**DOI:** 10.1186/s13148-022-01280-0

**Published:** 2022-05-17

**Authors:** Saori Aoki, Ken Higashimoto, Hidenori Hidaka, Yasufumi Ohtsuka, Shigehisa Aoki, Hiroyuki Mishima, Koh-ichiro Yoshiura, Kazuhiko Nakabayashi, Kenichiro Hata, Hitomi Yatsuki, Satoshi Hara, Takashi Ohba, Hidetaka Katabuchi, Hidenobu Soejima

**Affiliations:** 1 Division of Molecular Genetics and Epigenetics, Department of Biomolecular Sciences, Faculty of Medicine, Saga University, Saga, 849-8501 Japan; 2 Department of Obstetrics and Gynecology, Faculty of Life Sciences, Kumamoto University, Kumamoto, 860-8556 Japan; 3 Department of Pediatrics, Faculty of Medicine, Saga University, Saga, 849-8501 Japan; 4 Department of Pathology and Microbiology, Faculty of Medicine, Saga University, Saga, 849-8501 Japan; 5 Department of Human Genetics, Nagasaki University Graduate School of Biomedical Sciences, Nagasaki, 852-8523 Japan; 6 Department of Maternal-Fetal Biology, National Research Institute for Child Health and Development, Tokyo, 157-8535 Japan

**Keywords:** Placental mesenchymal dysplasia, Genomic imprinting, DNA methylation, Differentially methylated regions, Androgenetic/biparental mosaicism

## Abstract

**Background:**

Placental mesenchymal dysplasia (PMD) is a morphological abnormality resembling partial hydatidiform moles. It is often associated with androgenetic/biparental mosaicism (ABM) and complicated by Beckwith–Wiedemann syndrome (BWS), an imprinting disorder. These phenomena suggest an association between PMD and aberrant genomic imprinting, particularly of *CDKN1C* and *IGF2*. The existence of another type of PMD containing the biparental genome has been reported. However, the frequency and etiology of biparental PMD are not yet fully understood.

**Results:**

We examined 44 placental specimens from 26 patients with PMD: 19 of these were macroscopically normal and 25 exhibited macroscopic PMD. Genotyping by DNA microarray or short tandem repeat analysis revealed that approximately 35% of the macroscopic PMD specimens could be classified as biparental, while the remainder were ABM. We performed a DNA methylation analysis using bisulfite pyrosequencing of 15 placenta-specific imprinted differentially methylated regions (DMRs) and 36 ubiquitous imprinted DMRs. As expected, most DMRs in the macroscopic PMD specimens with ABM exhibited the paternal epigenotype. Importantly, the biparental macroscopic PMD specimens exhibited frequent aberrant hypomethylation at seven of the placenta-specific DMRs. Allelic expression analysis using single-nucleotide polymorphisms revealed that five imprinted genes associated with these aberrantly hypomethylated DMRs were biallelically expressed. Frequent aberrant hypomethylation was observed at five ubiquitous DMRs, including *GRB10* but not ICR2 or ICR1, which regulate the expression of *CDKN1C* and *IGF2*, respectively. Whole-exome sequencing performed on four biparental macroscopic PMD specimens did not reveal any pathological genetic abnormalities. Clinical and molecular analyses of babies born from pregnancies with PMD revealed four cases with BWS, each exhibiting different molecular characteristics, and those between BWS and PMD specimens were not always the same.

**Conclusion:**

These data clarify the prevalence of biparental PMD and ABM-PMD and strongly implicate hypomethylation of DMRs in the pathogenesis of biparental PMD, particularly placenta-specific DMRs and the ubiquitous *GRB10*, but not ICR2 or ICR1. Aberrant hypomethylation of DMRs was partial, indicating that it occurs after fertilization. PMD is an imprinting disorder, and it may be a missing link between imprinting disorders and placental disorders incompatible with life, such as complete hydatidiform moles and partial hydatidiform moles.

**Supplementary Information:**

The online version contains supplementary material available at 10.1186/s13148-022-01280-0.

## Background

Placental mesenchymal dysplasia (PMD) is a morphological abnormality that resembles partial hydatidiform moles but presents as placentomegaly and hydropic changes with vascularized edematous villi in the absence of abnormal trophoblastic proliferation. The condition can manifest as multiple thrombosis within the chorionic villi because of the tortuous vessels’ vulnerability to mesenchymal hyperplasia [1–4]. PMD is estimated to occur in 0.02% of all pregnancies [5], and roughly 80% of affected fetuses are female [3, 6]. Adverse pregnancy outcomes include high rates of fetal growth restriction, preterm delivery, and intrauterine fetal demise; neonatal outcomes include complications such as hematologic disorders (anemia and thrombocytopenia), liver tumors, and Beckwith–Wiedemann syndrome (BWS) [2, 3, 6].

With respect to the etiology of PMD, several studies have reported androgenetic/biparental mosaicism (ABM) in PMD specimens [7–10]. ABM can be caused by failed replication of the maternal genome after fertilization or by dispermy, which occurs when two haploid sperm cells fertilize one oocyte [10]. In PMD, androgenetic cells are distributed throughout the chorionic membrane, chorionic mesenchyme, stroma, and enlarged chorionic vessels [8, 11]. However, androgenetic cells are not distributed to the trophoblast, which contains only the biparental genome, leading to normal proliferation [8, 11].

The presence of ABM implies that disruption of genomic imprinting is involved in the etiology of PMD. Several findings have implicated the imprinted genes at 11p15.5, *CDKN1C* and *IGF2*, in the etiology of PMD. For example, BWS, which is caused by the disruption of *CDKN1C* or *IGF2* imprinting, has been found to occur in approximately 20% of PMD cases [6]; mosaicism of maternal deletion of 11p15.5 has been found in placentas with PMD [12]; and partial trisomy of 11p15.5 (two paternal copies and one maternal copy) has been found in an enlarged placenta with edematous villi [13]. Furthermore, mice with a null mutation of *Cdkn1c* and loss of *Igf2* imprinting have been reported to display placentomegaly and dysplasia, although human and mouse placentas have substantially different architecture [14].

In contrast to cases with ABM, several PMD cases have been found to be composed entirely of biparental cells and stromal cells positive for p57^KIP2^, which is encoded by *CDKN1C* [11, 15–18]. Therefore, there are two subsets of PMD: one involves ABM, in which imprinting disruption of *CDKN1C* or *IGF2* may be involved in the etiology; the other is biparental PMD, whose etiology is unknown. The frequency of biparental PMD among all PMD cases is also unknown.

In this study, we screened the genotypes of placental specimens obtained from 26 patients with PMD to investigate the frequency of biparental PMD. We also performed DNA methylation analysis of 51 imprinted differentially methylated regions (DMRs), including placenta-specific and ubiquitous DMRs. Further, we analyzed the allelic expression of several imprinted genes associated with aberrantly methylated DMRs. We also performed clinical and molecular analyses of babies born from pregnancies with PMD. Our results strongly suggest that imprinting disruption of several DMRs other than those for *CDKN1C* and *IGF2* is implicated in the pathogenesis of biparental PMD and may be a molecular link between PMD and other imprinting disorders.

## Results

### Approximately one-third of macroscopic PMD specimens show biparental genotype

Most of the placentas with PMD had two distinct areas: one with a macroscopically normal appearance and the other exhibiting macroscopic PMD characteristics (Additional file 3: Fig. S1). We tested 19 macroscopically normal specimens and 25 macroscopic PMD specimens for ABM via microarray or short tandem repeat analysis (Table 1; Additional file 3: Fig. S2). We found ABM in 64.0% (*n* = 16/25) of the macroscopic PMD specimens, which we denoted ABM-PMD, while the remaining 36.0% (*n* = 9/25) displayed normal biparental genotypes, which we denoted biparental-PMD (Table 1; Additional file 3: Figs. S2 and S3). Of the macroscopically normal placentas, just 21.1% (*n* = 4/19) exhibited ABM, which we denoted ABM-normal, while the other 78.9% (*n* = 15/19) displayed normal biparental genotypes, which we denoted biparental-normal (Table 1; Additional file 3: Figs. S2 and S3). The difference in ABM frequency between the macroscopic PMD (16/25, 64.0%) and macroscopically normal placentas (4/19, 21.1%) was significant (*p* = 0.005; chi-squared test). Further, ABM was more frequently isodisomic than heterodisomic (Table 1). We identified numerous copy number variations (CNVs) via microarray analysis, but none were obviously pathological (Additional file 3: Fig. S4). These findings support a link between aberrant imprinting due to ABM and the pathogenesis of PMD [7–10].

**Table 1:** Basic information on the placental mesenchymal dysplasia (PMD) cases analyzed in this study

ID	Macroscopically normal placental region	Macroscopic PMD region	Complications of BWS (BWSp score)	Molecular testing of baby’s PBL or CB	Baby’s sex (karyotype)	Conception	Gestational period	Delivery
PMD-001	ABM (iso)	Biparental	No	n/a	Female	Spontaneous	28w2d	Vaginal delivery
PMD-002	ABM (iso)	Biparental	No	No alteration	Female	Spontaneous	34w6d	Caesarean section
PMD-003	n/a	Biparental	No	No alteration	Female	Spontaneous	35w6d	Vaginal delivery
PMD-008	Biparental	ABM (iso)	No	n/a	Female	Artificial insemination	35w3d	Caesarean section
PMD-010	Biparental (8p partial trisomy)	ABM (hetero) (8p partial trisomy)	n/a	n/a	Female (46,XX)	Spontaneous	16w4d	Artificial abortion
PMD-012	Biparental	ABM (iso)	No	No alteration	Female (46,XX)	Spontaneous	31w2d	Caesarean section
PMD-016*	n/a	ABM (iso)	No	No alteration	Male	Spontaneous	27w5d	Caesarean section
PMD-018	n/a	ABM (iso)	n/a	n/a	Female	No information	15w0d	Artificial abortion
PMD-020	Biparental	Biparental	Yes (5)	ICR2-LOM	Male	Spontaneous	26w0d	Caesarean section
PMD-021	ABM (hetero)	ABM (hetero)	No	No alteration	Male	Spontaneous	37w2d	Vaginal delivery
PMD-022	Biparental	ABM (iso)	Yes (6)	No alteration	Female (46,XX)	Spontaneous	33w0d	Caesarean section
PMD-023	Biparental	ABM (iso)	No	n/a	Female (46,XX)	Spontaneous	37w3d	Vaginal delivery
PMD-024**	ABM (iso)	Biparental	No	n/a	Female (46,XX)	Spontaneous	36w6d	Vaginal delivery
PMD-028**	Biparental	n/a	No	n/a	Female (46,XX)	Spontaneous	36w6d	Vaginal delivery
PMD-029**	Biparental	ABM (iso)	No	n/a	Female (46,XX)	Spontaneous	36w1d	Vaginal delivery
PMD-030*	n/a	biparental	No	n/a	Male	Spontaneous	29w4d	Caesarean section
PMD-033**	Biparental	ABM (iso)	No	n/a	Female (46,XX)	Spontaneous	37w1d	Vaginal delivery
PMD-034	Biparental	Biparental	No	n/a	Female (46,XX)	Frozen–thawed Embryo transfer	30w5d	Caesarean section
PMD-035	Biparental	ABM (iso)	No	n/a	Female	Spontaneous	36w5d	Caesarean section
PMD-039	n/a	Biparental	No	No alteration	Female	Spontaneous	38w0d	Caesarean section
PMD-041	n/a	ABM (hetero)	n/a	n/a	No information	Spontaneous	16w6d	Spontaneous abortion
PMD-042	Biparental^†^	ABM (iso)^†^	No (3)	No alteration	Female (46,XX)	Spontaneous	35w2d	Vaginal delivery
PMD-043	Biparental^†^	ABM (iso)^†^	No	No alteration	Female	Spontaneous	38w2d	Vaginal delivery
PMD-045	Biparental^†^	ABM (iso)^†^	n/a	n/a	No information	In vitro fertilization, Frozen–thawed Embryo transfer	14w0d	Artificial abortion
PMD-bws022	n/a	Biparental	Yes (13)	patUPD	Female (46,XX)	Spontaneous	35w3d	Caesarean section
PMD-bws027	Biparental^†^	ABM (iso)^†^	Yes (5)	ABM (iso)^†^	Male^‡^ (46,XY[17]/46,XX[2])	Spontaneous	26w2d	Caesarean section

ABM, androgenetic/biparental mosaicism; BWS, Beckwith–Wiedemann syndrome; BWSp: Beckwith–Wiedemann spectrum; CB; cord blood; hetero, heterodisomy; ICR2-LOM, loss of methylation at imprinting control region 2; iso, isodisomy; patUPD, segmental paternal uniparental disomy of chromosome 11p; PBL, peripheral blood leukocytes; n/a: not available or not analyzed

^*^Macroscopic PMD lesion occupied the whole placenta

^**^Cystic region shrank during pregnancy in PMD-024, -028, -029, and -033; in PMD-028, only the macroscopically normal region was available for the analyses

^†^Analyzed via short tandem repeats

^‡^Early neonatal death

### Aberrantly hypomethylated imprinted DMRs in biparental-PMD specimens

The existence of biparental-PMD specimens prompted us to analyze the DNA methylation status of imprinted DMRs (Additional file 3: Fig. S2). We analyzed biparental-normal (*n* = 11), biparental-PMD (*n* = 8), and ABM-PMD (*n* = 6) specimens, using quantitative bisulfite pyrosequencing, at 15 placenta-specific DMRs, which have been verified as gametic maternally methylated DMRs in several previous studies [19–21]. We also analyzed 36 ubiquitous DMRs and compared the results with those for normal placenta specimens. We defined aberrant hypomethylation as less than the mean for normal placentas minus two standard deviations (SDs), and aberrant hypermethylation as more than the mean for normal placentas plus two SDs.

In the ABM-PMD specimens, most of the DMRs were aberrantly methylated: The maternally methylated DMRs were aberrantly hypomethylated, while the paternally methylated DMRs were aberrantly hypermethylated (Table 2, Additional file 1: Table S1). These results were consistent with ABM. In the biparental-PMD and biparental-normal specimens, we focused on identifying the DMRs that were most frequently affected. For this purpose, we counted the number of aberrantly hypomethylated DMRs, which we defined as DMRs that were aberrantly hypomethylated but not aberrantly hypermethylated in more than half of the specimens (i.e., four or more of the biparental-PMD specimens, or six or more of the biparental-normal specimens). In the biparental-PMD specimens, seven of 15 (46.7%) placenta-specific DMRs (*MCCC1*, *AIM1*, *AGBL3*, *GLIS3*, *FAM196A*, *N4BP2L1*, and *FAM20A*) were aberrantly hypomethylated in more than half of the specimens, whereas five of 25 (20.0%) gametic maternally methylated ubiquitous DMRs (*PPIEL*, *NAP1L5*, *GRB10*, *NESPAS*-*GNASXL*, and *WRB*) were aberrantly hypomethylated in more than half of the specimens (Tables 2 and 3, Additional file 1: Table S1). In the biparental-normal specimens, there were no placenta-specific DMRs showing aberrant hypomethylation in more than half of the specimens, whereas one ubiquitous DMR, *WRB*, showed aberrant hypomethylation in 10 of 11 specimens. These results indicated that the gametic maternally methylated DMRs were aberrantly hypomethylated according to the progress of the macroscopic and genetic changes in the PMD specimens, and that placenta-specific DMRs were more affected in the PMD specimens.

**Table 2:**
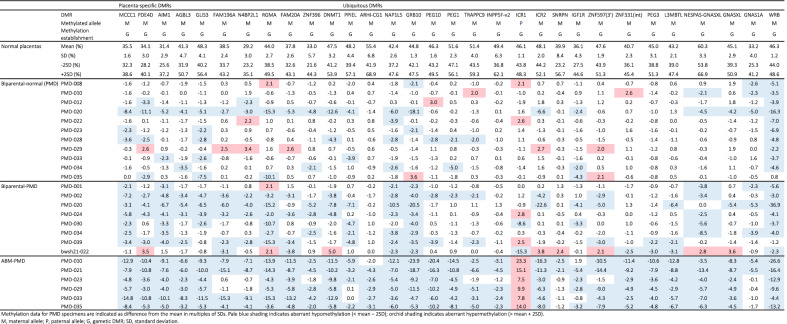
The number of aberrantly hypomethylated DMRs in more than half of the biparental-normal and biparental-PMD specimens

**Table 3:** The number of aberrantly hypomethylated DMRs in more than half of the biparental-normal and biparental-PMD specimens

	Placenta-specific DMRs	Ubiquitous DMRs
Biparental-normal specimens*	0/15	1/25
Biparental-PMD specimens**	7/15	5/25

^*^More than half of biparental-normal specimens: ≥ 6

^**^More than half of biparental-PMD specimens: ≥ 4

It was intriguing that among the ubiquitous DMRs, *GRB10* was aberrantly hypomethylated in all eight biparental-PMD specimens, and *NAP1L5* and *WRB* were in seven of them (Table 2). On the other hand, two DMRs critical for BWS pathogenesis, ICR1 and ICR2, were not frequently affected in the biparental-PMD specimens: ICR1 was hypermethylated and hypomethylated in two specimens each, and ICR2 was hypermethylated and hypomethylated in one and two specimens, respectively (Table 2). Furthermore, ICR1-related somatic DMRs, such as *H19*-promoter, *IGF2*-DMR0, and *IGF2*-DMR2, were barely affected in the biparental-PMD specimens (Additional file 1: Table S1).

### Biallelic expression of imprinted genes associated with placenta-specific DMRs in biparental-PMD specimens

**Fig. 1:**
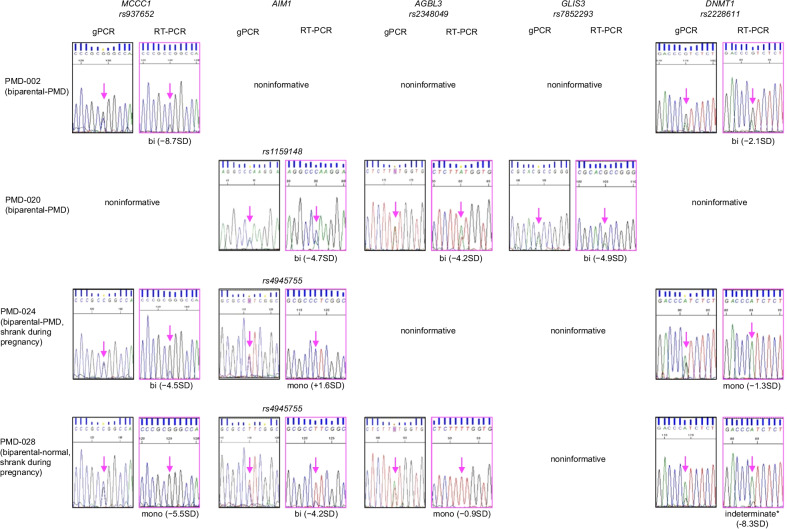
Biallelic expression of placenta-specific imprinted genes in specimens of biparental placental mesenchymal dysplasia (PMD). Using single-nucleotide polymorphisms (SNPs), we screened the genotypes of imprinted genes via polymerase chain reaction with genomic DNAs (gPCR) and examined their allelic expression via reverse-transcription PCR (RT-PCR). Two biparental-PMD specimens (PMD-002 and PMD-020) showed biallelic expression. Specimen PMD-024 was a biparental-PMD specimen in which the cystic region had shrunk during pregnancy; this specimen showed biallelic expression of one gene and monoallelic expression of two genes. Specimen PMD-028 was a biparental-normal specimen in which the cystic region had also shrunk during pregnancy; in this specimen, one gene exhibited biallelic expression, two exhibited monoallelic expression, and one was indeterminate (*) because of very low expression of the maternal allele in some of the normal placental samples we used as controls (Additional file 3: Fig. S5). Arrows indicate the positions of the SNPs. bi: biallelic expression; mono: monoallelic expression. Differences in DNA methylation levels between the normal specimens and the PMD specimens are indicated in parentheses. Since the analyses were performed on additional specimens excised from placental tissues, the methylation levels differ from those shown in Table 2, which refer to the original specimens

Next, we analyzed whether the aberrant hypomethylation at placenta-specific DMRs influenced the allelic expression of the imprinted genes associated with each DMR. Using single-nucleotide polymorphism (SNP)-based genotype screening, we identified four specimens—three biparental-PMD (PMD-002, PMD-020, and PMD-024) and one biparental-normal (PMD-028)—as informative (heterozygous) for at least two of five genes: *MCCC1*, *AIM1*, *AGBL3*, *GLIS3*, and *DNMT1* (Fig. 1). PMD-002 was informative for *MCCC1* and *DNMT1*; PMD-020 was informative for *AIM1*, *AGBL3*, and *GLIS3*; PMD-024 was informative for *MCCC1*, *AIM1*, and *DNMT1*; and PMD-028 was informative for *MCCC1*, *AIM1*, *AGBL3*, and *DNMT1*. The cystic region in specimens PMD-002 and PMD-020 continued to term, as often occurs, but in PMD-024 and PMD-028 it shrank during pregnancy, indicating that the phenotypes of these cases were milder than those of PMD-002 and PMD-020. We confirmed that all five genes were preferentially expressed from the paternal allele in normal placentas (Additional file 3: Fig. S5). However, we observed biallelic expression of all five genes associated with aberrant hypomethylation in specimens PMD-002 and PMD-020, both of which were biparental-PMD. In PMD-024, we found biallelic expression of one gene associated with aberrant hypomethylation and monoallelic expression of two genes associated with methylation within the normal range. In PMD-028, a biparental-normal specimen, there was biallelic expression of one gene associated with aberrant hypomethylation and monoallelic expression of two genes, one of which was associated with aberrant hypomethylation and the other with normal-range methylation. The allelic expression of *DNMT1* in PMD-028 was indeterminate because of very low expression levels of the maternal alleles in some of the normal placenta samples we used as controls (Additional file 3: Fig. S5). Although we could not evaluate the total expression levels of these genes because of the instability of the quantitative reverse-transcription polymerase chain reaction (RT-PCR) analysis due to poor RNA quality, these data strongly suggest that biallelic expression of placenta-specific imprinted genes is correlated with aberrant hypomethylation and with the typical phenotype (PMD-002 and PMD-020) rather than milder phenotype (PMD-024 and PMD-028). Therefore, disruption of imprinting, especially placenta-specific imprinted genes, may be involved in the pathogenesis of PMD.

### Putative pathogenic variants not detected in biparental-PMD specimens

Pathological variants of zinc finger protein genes such as *ZFP57* and *ZNF445* and of genes related to the subcortical maternal complex such as *NLRP2*, *NLRP5*, *NLRP7*, and *KHDC3L* have been identified in cases of multilocus imprinting disturbances (MLIDs) and recurrent hydatidiform moles (RHMs) [22]. Whole-exome sequencing (WES) of four biparental-PMD specimens (PMD-001, PMD-002, PMD-003, and PMD-bws022) and one biparental-normal specimen (PMD-008) failed to reveal any pathological variants of the above genes or any others, irrespective of zygosity (data not shown).

### Molecular investigation reveals babies with Beckwith–Wiedemann syndrome born following PMD-complicated pregnancies

Twenty-two babies were born from 26 patients with PMD (Table 1 and 4). Seventeen were female and five were male, one of which (PMD-bws027) exhibited mosaicism of the sex chromosomes (Table 1). The male–female ratio was consistent with that of previous reports [3, 6]. We examined the clinical features of these babies and scored them according to the Beckwith–Wiedemann spectrum (BWSp) scoring system [23]. We then examined 12 of these babies for the main known causative alterations characteristic of BWS, including gain of methylation at ICR1, loss of methylation at ICR2 (ICR2-LOM), paternal uniparental disomy of chromosome 11 (patUPD), *CDKN1C* pathogenic variant, and CNVs of chromosome 11p. Of the 12 babies from ABM-PMD pregnancies, we found that two (PMD-022 and PMD-bws027) could be diagnosed with classic BWS (BWSp score ≥ 4). One of these showed no alterations, while the other exhibited ABM, indicating paternal uniparental diploidy (Table 4). We also found that one case (PMD-042), who had a BWSp score of 3, showed no alterations and was not diagnosed with BWS. However, of nine babies from pregnancies exhibiting biparental-PMD, two could be diagnosed with classic BWS. One of these displayed ICR2-LOM (PMD-020) and the other exhibited patUPD limited to 11p (PMD-bws022). The frequency of BWS in babies born from pregnancies with PMD (18.2%, 4/22) was similar to that reported previously [6].

**Table 4:** Molecular analyses of BWS alterations in babies born from PMD-complicated pregnancies

PMD status	Number of babies	Number of babies with BWSp score ≥ 4	Molecular testing
ABM-PMD	12	2	ABM (paternal uniparental diploidy), no alteration
Biparental-PMD	9	2	ICR2-LOM, patUPD
Biparental-normal*	1	0	

*ICR2-LOM* loss of methylation at imprinting control region 2, *patUPD* segmental paternal uniparental disomy of chromosome 11p

*PMD-028, in which the cystic region shrank during pregnancy

## Discussion

Our study is the first to assess the incidence of ABM-PMD and biparental PMD by splitting the affected placental specimens into macroscopically normal and macroscopic PMD tissue samples. The presence of ABM even in macroscopically normal tissue from placentas with PMD, along with its significantly greater prevalence in macroscopic PMD tissue than in macroscopically normal tissue, supports the existence of an association between aberrant imprinting and PMD. The fact that ABM was more frequently isodisomic than heterodisomic is consistent with the established evidence indicating that it is more often caused by failed replication of the maternal genome following normal fertilization than by dispermy [24].

The most important findings in this study were aberrant hypomethylation at imprinted DMRs in biparental-PMD specimens, especially at placenta-specific DMRs, along with altered biallelic expression of associated imprinted genes. We found that the frequency of aberrant hypomethylation in placenta-specific DMRs was higher than that in ubiquitous DMRs. Biallelic expression of five associated imprinted genes occurred in biparental-PMD with the typical phenotype more commonly than in the milder phenotype. These results strongly suggest that aberrant imprinting is involved in PMD pathogenesis. While polymorphic imprinting of placenta-specific DMRs has been reported previously [20, 21, 25], our results suggest a relatively minor contribution of polymorphic imprinting and a greater contribution of aberrant hypomethylation to PMD pathogenesis. We also detected frequently aberrant methylation in several gametic maternally methylated ubiquitous DMRs in our biparental-PMD specimens. Notably, ICR2 and ICR1 were not included in this group, and *GRB10* was aberrantly hypomethylated in all biparental-PMD specimens. The DMR associated with *GRB10*, which is an imprinted gene that encodes a growth inhibitor, is maternally methylated in the human placenta, and the maternal allele is expressed [26]. Since DMR methylation and expression levels are positively correlated [27], reduced expression is assumed in biparental-PMD specimens. In addition, maternal deletion of *GRB10* has been reported in the enlarged cystic villi of placentas with biparental PMD and the enlarged placentas of maternal knockout mice [28, 29]. These results suggest that hypomethylation at *GRB10* is involved in the pathogenesis of biparental PMD. *NAP1L5* and *WRB* were also frequently aberrantly hypomethylated in biparental-PMD specimens, but these genes may not be involved in the pathogenesis of biparental PMD for the following reasons. Mice with two paternal copies of the chromosomal region including *NAP1L5* and other imprinted genes showed normal-sized placentas [30]. *WRB* is biallelically expressed in 10 human tissues [31], and the methylation status of several CpGs in and around the DMR analyzed in this study is not correlated with gene expression levels in human placentas [32]. Together, our results lead us to surmise that a switch from the paternal to the maternal epigenotype at certain DMRs, including placenta-specific DMRs and at least one ubiquitous DMR (*GRB10*), is strongly linked to PMD pathogenesis.

In contrast to the aberrant hypomethylation of a subset of DMRs in biparental-PMD specimens, the majority of maternally methylated ubiquitous DMRs and placenta-specific DMRs were severely hypomethylated in RHMs from females with *NLRP7* or *KHDC3L* mutations [33, 34]. This suggests that the difference in the incidence and degree of aberrant hypomethylation of DMRs is critical for the pathogenesis of either PMD or RHMs.

Nearly all the aberrantly hypomethylated DMRs we identified were at maternally methylated DMRs. Hypomethylation is thought to occur after fertilization, because the DMR methylation does not reach the minimum level of 0%, suggesting mosaicism of hypomethylated and normally methylated cells. Maternal methylation of ubiquitous DMRs is protected from demethylation between fertilization and implantation and preserved post-implantation by DNMT1, which is recruited via ZFP57, ZFP445, and TRIM28 [35]. In the case of human placenta-specific DMRs, however, the mechanism that maintains maternal methylation remains poorly understood. It has been suggested that site-specific exclusion of DNA methyltransferases or selective recruitment of demethylation-related factors is critical to the mechanisms of methylation maintenance [21]. DMR hypomethylation in placentas with biparental PMD seems to be caused by a disruption of these mechanisms, although the etiology of this disruption is unknown.

We detected several CNVs in our analyses, but none of them were pathological or affected any of the DMRs analyzed in this study. Further, our WES analysis did not identify any pathological variants of maternal effect genes, including those linked to MLIDs and RHMs. Recently, missense variants of three genes—*NLRP2* (p.Thr516Ala), *NLRP7 (*p.Val319Ile), and *ATRX* (p.Arg808Gln)—in a single case of biparental PMD were reported [17]. However, given the interpretation of these variants as benign or likely benign on ClinVar (https://www.ncbi.nlm.nih.gov/clinvar/), the pathogenicity of these variants is probably minimal. Therefore, PMD seems to be primarily caused by epigenetic rather than genetic factors, although because our WES sample size was small and we did not conduct a WES analysis of mothers with pregnancies complicated by PMD, we cannot completely rule out the involvement of the maternal effect genes mentioned above or of other genetic factors.

Similar to a previous report [6], 18% of the babies in our study that were born from pregnancies with PMD had BWS. The molecular characteristics of babies with BWS and of PMD specimens were not always the same, suggesting that the cells of origin in BWS and PMD also differ. In ABM-PMD and biparental PMD, androgenetic cells and aberrantly hypomethylated cells may arise at the first cleavage of the zygote [8–10] and during the preimplantation period, respectively. The molecular defects that are causative of BWS, such as patUPD and ICR2-LOM, also arise during the preimplantation period. It is possible that differences in the extent of the uniparental disomic (UPD) region or aberrant hypomethylation of DMRs is one of the critical factors determining cell fate, differentiation into extraembryonic tissue or embryonic tissue, or later retention or elimination in either tissue type.

Two cases of PMD occurred in pregnancies that resulted from assisted reproductive technology (ART). It is known that the risk of imprinting disorders increases in babies conceived via ART [36, 37]. However, thus far, only one ABM-PMD pregnancy with twins resulting from in vitro fertilization has been reported [38]. Therefore, specimens from more PMD cases should be collected and analyzed to improve our understanding of the relationship between PMD and ART.

## Conclusions

The data obtained in this study strongly implicate DMR hypomethylation in the pathogenesis of biparental PMD, particularly hypomethylation of placenta-specific DMRs and the ubiquitous *GRB10*, but not of ICR2 and ICR1. Therefore, both ABM-PMD and biparental PMD are imprinting disorders, which may constitute a missing link between imprinting disorders in liveborn children and placental disorders that are incompatible with life, such as partial and complete hydatidiform moles (including RHMs). Since the functions of placenta-specific imprinted genes have not yet been resolved [20, 21], functional analysis of these genes should be conducted to elucidate their relationship to PMD pathogenesis. In addition, whole-genome methylation analysis beyond imprinted DMRs, exploration of the genetic origins of PMD by conducting WES of more placental samples and mothers, and whole-genome sequencing analyses are important for further clarifying the pathogenesis of PMD. In the future, it may become possible to diagnose PMD via noninvasive prenatal testing, based on specific epigenomic or genomic abnormalities, which should usefully inform clinical diagnostics and pregnancy care.

## Methods

### Placental tissue

We collected data on 49 cases of PMD from across Japan [39]. Of these, 26 placentas from patients with PMD (fetal sex: 19 female, 5 male, 2 unspecified; average gestation: 33 weeks 6 days ± 28 days, except for four abortions) were available for nucleic acid extraction and molecular analyses (Table 1). All PMD cases were diagnosed by at least two experts in placental pathology, according to the specific pathological features specified by Lokan et al. [1]. Most of the placentas with PMD displayed two distinct areas: an area with a macroscopically normal appearance and an area exhibiting characteristic macroscopic PMD (Additional file 3: Figure S1). In four of the specimens (PMD-024, PMD-028, PMD-029, and PMD-033), the cystic PMD region shrank during the pregnancy, but a macroscopic PMD region was still present at birth for all specimens except PMD-028. In this specimen, only a macroscopically normal sample was available for the analyses. Twenty normal placentas were used as controls (fetal sex: 10 female, 10 male; average gestation: 36 weeks 5 days ± 14 days).

### Nucleic acid extraction

We extracted genomic DNA from 19 macroscopically normal specimens, 25 macroscopic PMD specimens, and 20 control placentas using the QIAamp DNA Mini Kit, following the manufacturer’s instructions (QIAGEN, Hilden, Germany). Total RNA was extracted from placental tissue using ISOGEN II according to the manufacturer’s instructions (Nippon Gene, Tokyo, Japan).

### DNA microarray analysis

We used the Genome-Wide Human SNP Array 6.0 (Affymetrix, Santa Clara, CA, USA) and the CytoScan HD Array (Affymetrix) to investigate ABM and CNVs. The genotypes generated by the SNP array were subjected to Genotyping Console 4.0 analysis (Affymetrix), and the copy number state and allele ratios were analyzed using Nexus Copy Number software 6.0 (BioDiscovery, Hawthorne, CA, USA). The genotypes, CNVs, and allele ratios from the CytoScan array were analyzed using the CytoScan Chromosome Analysis Suite, version 2.1 (Affymetrix). The genomic positions of the SNPs from the SNP Array and CytoScan corresponded to NCBI36/hg18 and GRCh37/hg19, respectively.

### Short tandem repeat marker analysis

For the quantitative analyses, we used 12 short tandem repeat markers (tetranucleotide repeat markers) on chromosomes 11, 14, 15, and 16 to investigate ABM as previously described [40, 41]. These markers were amplified and separated via electrophoresis using an Applied Biosystems 3130 genetic analyzer (Applied Biosystems, Foster City, CA, USA). We then quantitatively analyzed the data using the Peak Scanner 2 software (Applied Biosystems).

### Methylation analysis of imprinted DMRs via bisulfite pyrosequencing

Genomic DNA (500 ng) was subjected to bisulfite conversion using the EZ DNA Methylation Kit (Zymo Research, Irvine, CA, USA). We analyzed the methylation status of imprinted DMRs via bisulfite pyrosequencing using the PyroMark Q24 pyrosequencing instrument (QIAGEN) according to the manufacturer’s instructions. To validate the quantitative capability of bisulfite pyrosequencing methylation analysis, we evaluated all the primer sets we designed using varying mixtures of unmethylated and fully methylated control DNA (0%, 25%, 50%, 75%, or 100% methylated DNA), as previously described [42, 43]. All primers used for the methylation analyses are listed in Additional file 2: Table S2.

### Genotyping and expression analysis of *MCCC1, AIM1, AGBL3, GLIS3,* and *DNMT1*

We used additional excised specimens from placental tissues with PMD for our genotyping and expression analysis. We used SNPs to screen informative samples and analyze allelic expression of the following genes: rs937652 in exon 1 of *MCCC1*; rs4945755 in exon 1 of *AIM1*; rs1159148 in exon 2 of *AIM1*; rs2348049 in exon 4 of *AGBL3*; rs7852293 in exon 1 of *GLIS3*; and rs2228611 in exon 17 of *DNMT1*. We performed the genotyping and allelic expression analysis via PCR followed by Sanger sequencing using the Applied Biosystems 3130 genetic analyzer. We treated the RNA with RNase-free DNase I (TAKARA, Tokyo, Japan) and performed reverse transcription using random primers and ReverTra Ace (Toyobo, Osaka, Japan). All primers used for the genotyping and allelic expression analysis are listed in Additional file 2: Table S2.

### Whole-exome sequencing

We performed WES on four biparental-PMD specimens (PMD-001, PMD-002, PMD-003, and PMD-bws022) and one biparental-normal specimen (PMD-008). We sequenced enriched libraries prepared using SureSelect Human All Exon V4 + UTRs (Agilent Technologies, Santa Clara, CA, USA) using the SOLiD 5500xl 50 bp + 25 bp paired-end procedure (Thermo Fisher Scientific, Waltham, MA, USA). We processed the read data we obtained using an in-house workflow [44] to align them to the hg19 human reference genome with NovoAlignCSMPI version 1.02.03 (Novocraft Technologies, Petaling Jaya, Selangor, Malaysia). We also used the read data to call single-nucleotide variations and small insertions and deletions of bases using the UnifiedGenotyper program in the Genome Analysis Toolkit version 2.3 [45]. We omitted common variations by filtering out variants that had alternative allele frequencies (AAF) greater than the threshold in any of the following databases: the October 2014 release of the 1000 Genomes Project (AAF > 0.5%), the National Heart, Lung, and Blood Institute Exome Sequencing Project ESP6500SI-V2 (AAF > 0.5%), the Human Genetic Variation Database [46] version 1.42 (AAF > 0.5%), and the Complete Genomics 46 genomes database (AAF > 2%). We used GENCODE version 19 to classify deleterious variants that were nonsynonymous, had gained or lost stop codons, were within 2 bp of exon–intron boundaries, or contained in-frame or frameshift insertions and deletions. We also omitted variations within regions tagged as genomic segmental duplications in the University of California Santa Cruz Genome Browser.

### Statistical analysis

We used chi-squared tests to compare the frequency of ABM between macroscopically normal and macroscopic PMD specimens. We considered *p* values less than 0.05 to be statistically significant.

## Supplementary Information

**Additional file 1**. Supplementary Table S1.

**Additional file 2**. Supplementary Table S2.

**Additional file 3**. Supplementary Figures.

## Data Availability

Data generated or analyzed during this study, except for the WES data, are included in this published article and its supplementary information files. The CNV and WES datasets are not publicly available because no positive data were obtained, but they are available from the corresponding author upon reasonable request.

## Abbreviations

AAF: Alternative allele frequencies
ABM: Androgenetic/biparental mosaicism
ART: Assisted reproductive technology
BWS: Beckwith–Wiedemann syndrome
BWSp: Beckwith–Wiedemann spectrum
CNV: Copy number variation
DMR: Differentially methylated region
ICR2-LOM: Loss of methylation at ICR2
MLID: Multilocus imprinting disturbance
patUPD: Paternal uniparental disomy of chromosome 11
PCR: Polymerase chain reaction
PMD: Placental mesenchymal dysplasia
RHMs: Recurrent hydatidiform moles
RT-PCR: Reverse-transcription polymerase chain reaction
SD: Standard deviation
SNP: Single-nucleotide polymorphism
UPD: Uniparental disomy
WES: Whole-exome sequencing
