# Supplementary material for: Aberrant hypomethylation at imprinted differentially methylated regions is involved in biparental placental mesenchymal dysplasia
Source: Clin Epigenetics. 2022 May 17;14:64. doi: 10.1186/s13148-022-01280-0 (PMC9115938; doi:10.1186/s13148-022-01280-0)
Supplement: Supplementary file 3 — Additional file 3. Supplementary Figures. [file 13148_2022_1280_MOESM3_ESM.pdf]

Fig. S1

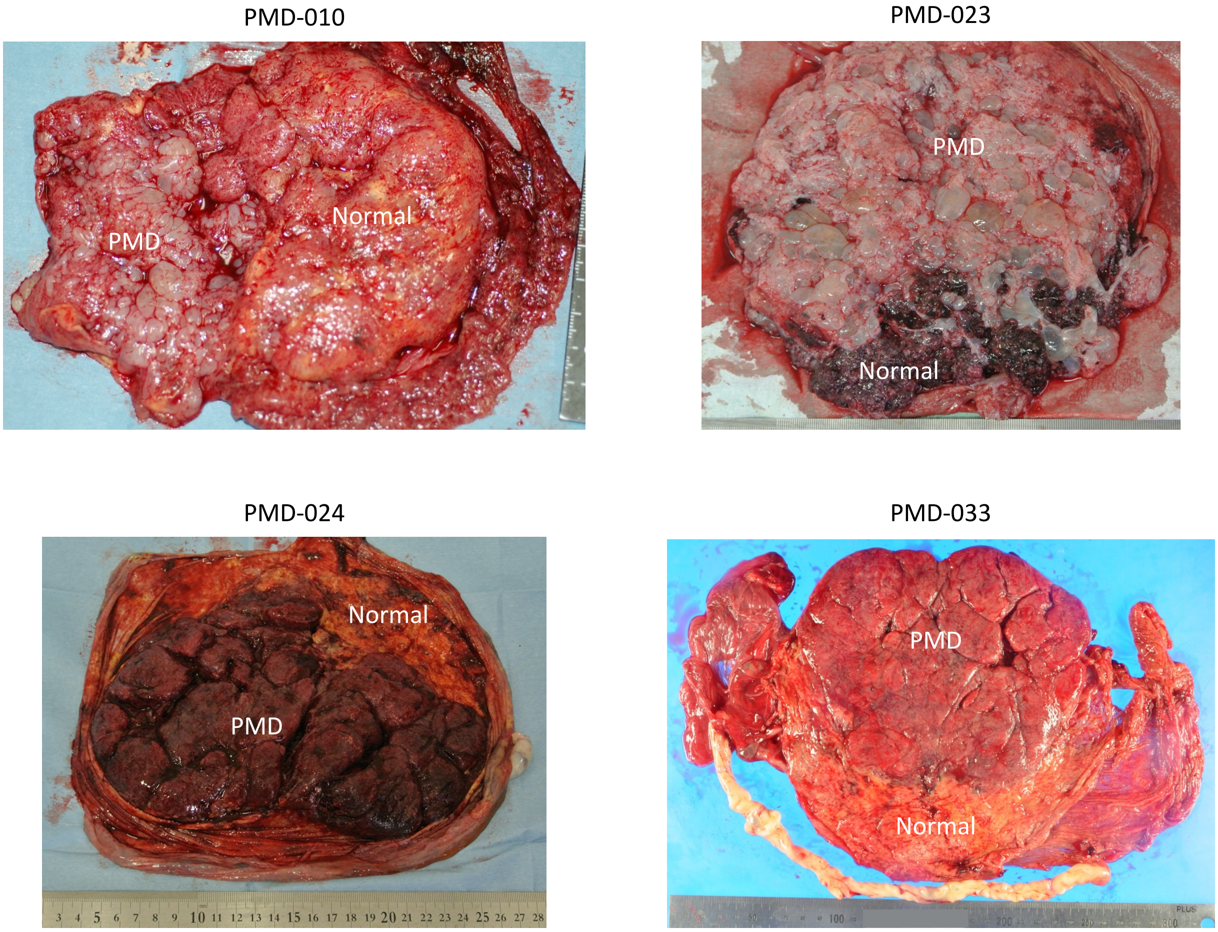

Supplementary Fig. S1. Representative macroscopic findings of placentas with placental mesenchymal dysplasia (PMD)

Maternal surfaces are shown. All placentas in this figure have two distinct areas: an area with a macroscopically normal appearance (normal) and an area exhibiting macroscopic PMD characteristics (PMD).

Fig. S2

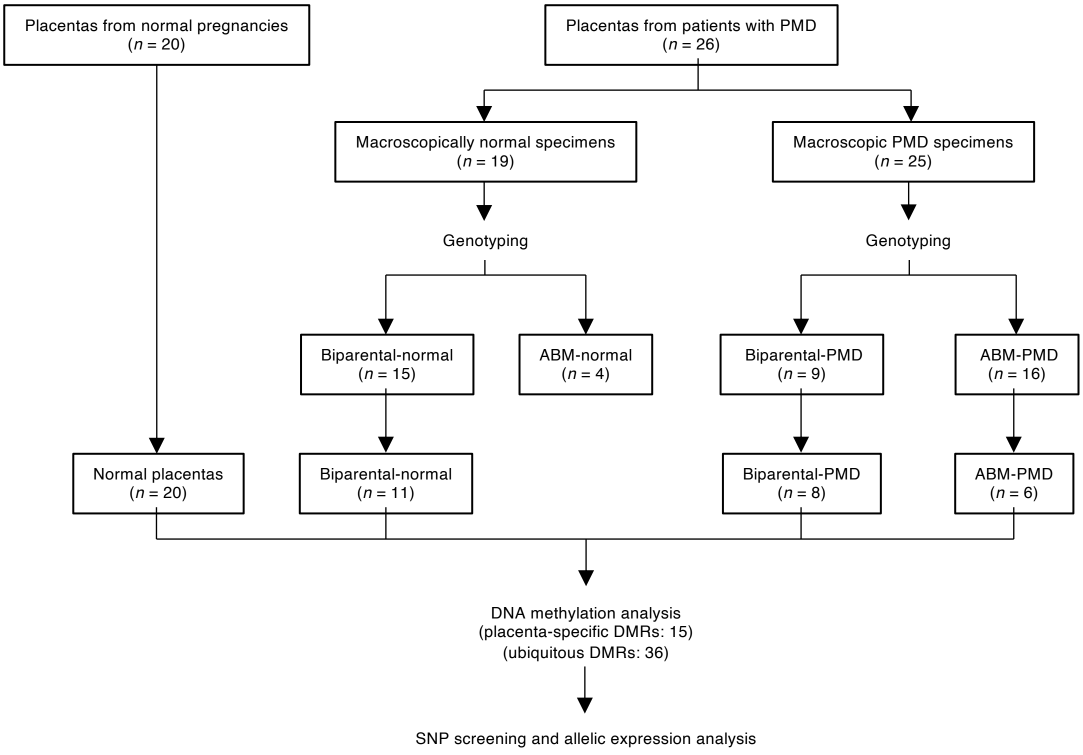

Supplementary Fig. S2. Experimental flowchart

From 26 placentas with placental mesenchymal dysplasia (PMD), we obtained 19 macroscopically normal specimens and 25 macroscopic PMD specimens for genotyping via single-nucleotide polymorphism (SNP) arrays or short tandem repeat markers. We then subjected randomly selected PMD samples from each genotype group (including the normal placental samples) to comprehensive DNA methylation analysis. Finally, we performed SNP screening and allelic expression analysis of five placenta-specific genes. ABM, androgenetic/biparental mosaicism; DMR, differentially methylated region.

Fig. S3

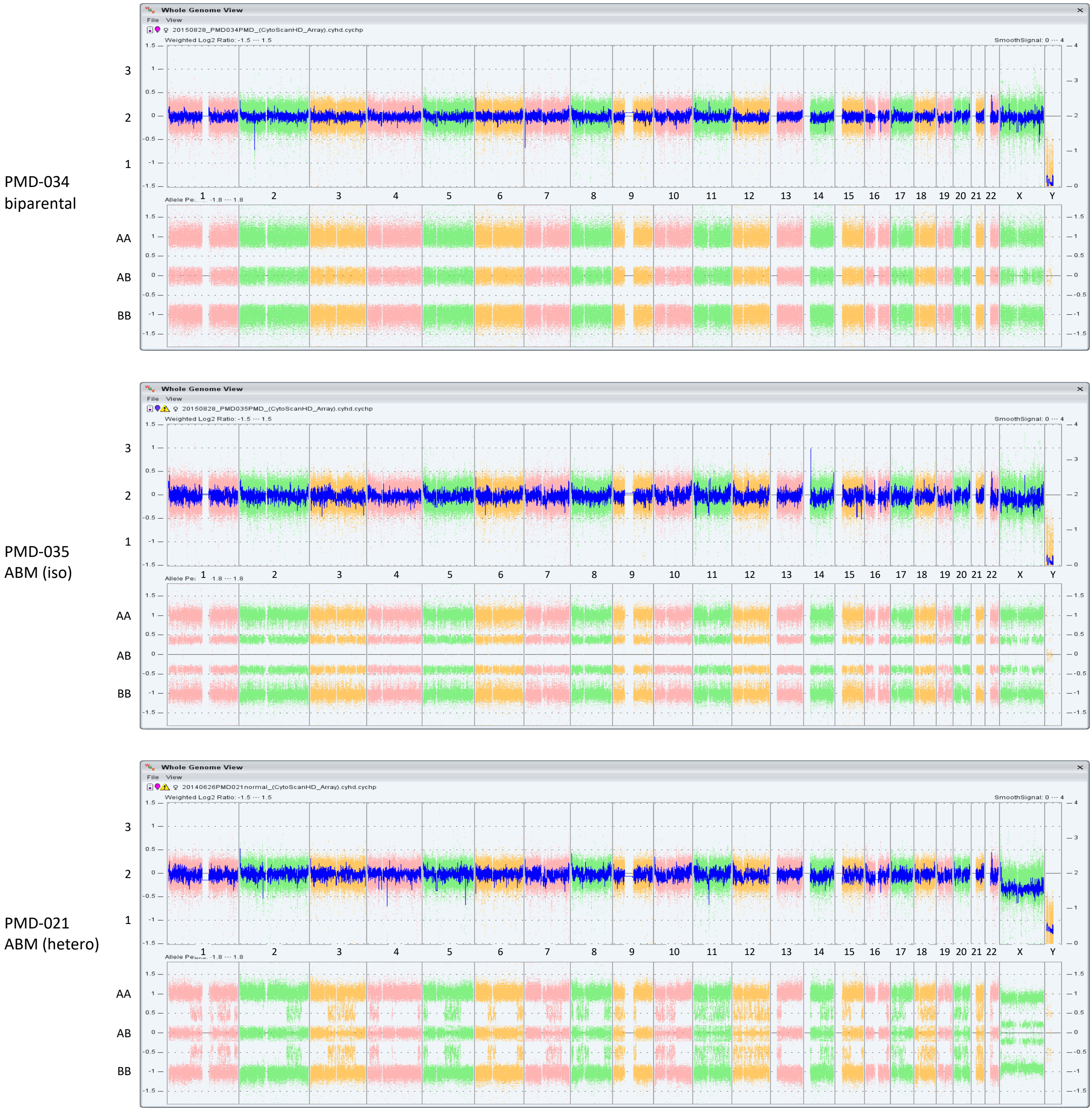

Supplementary Fig. S3. Representative whole-genome views from CytoScan of macroscopic placental mesenchymal dysplasia (PMD) specimens

The upper graph in each whole-genome view presents the weighted $\log_2$ ratios of the copy-number state, and the lower graph presents the allele differences. PMD-034 exhibited a normal biparental genotype with two normal copies. PMD-035 exhibited isodisomic (iso) androgenetic/biparental mosaicism (ABM), and PMD-021 displayed heterodisomic (hetero) ABM. The data were analyzed using the Affymetrix Chromosome Analysis Suite 2.1.

Fig. S4

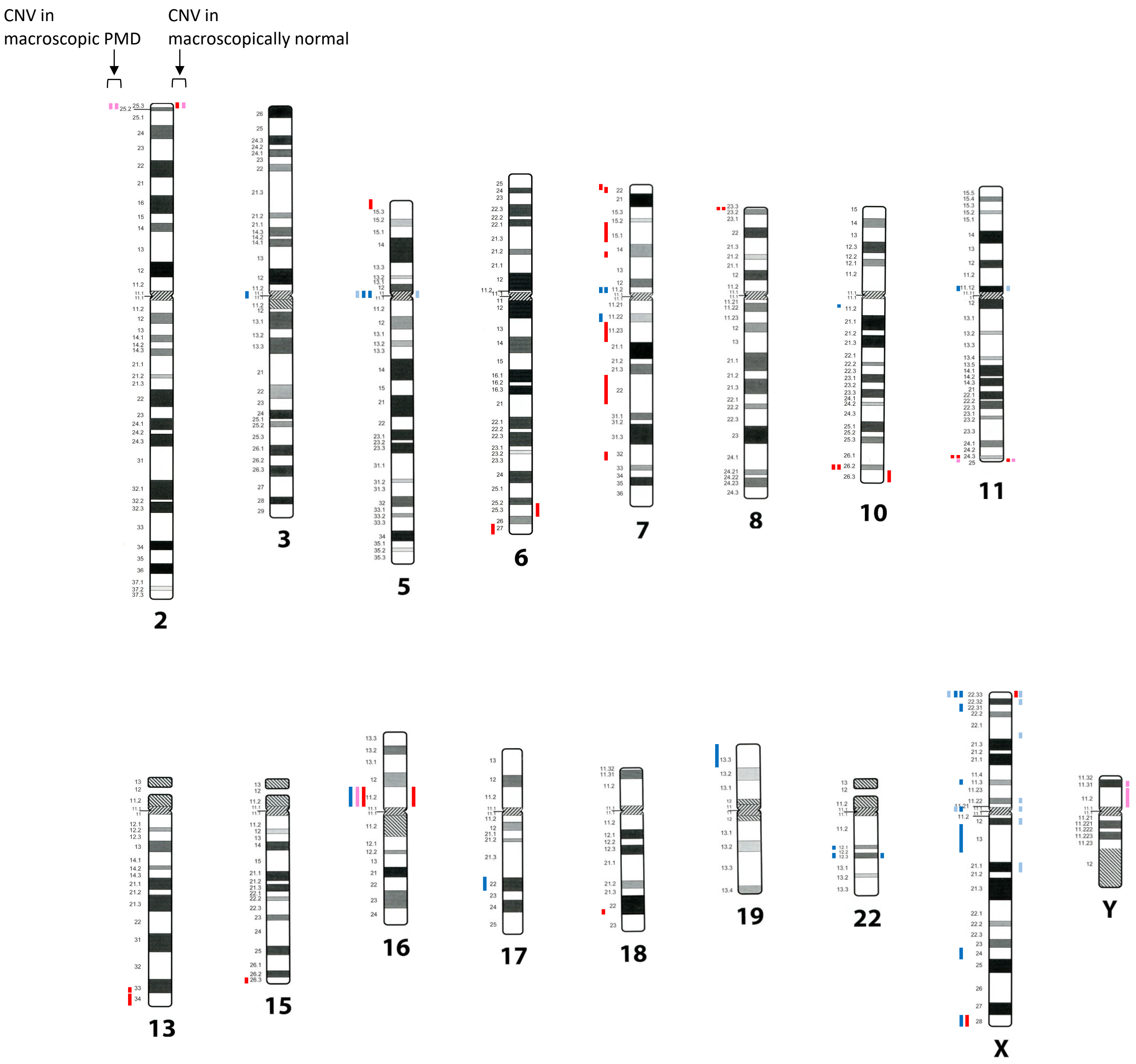

Supplementary Fig. S4. Summary of SNP array analysis of PMD specimens

CNVs in macroscopic PMD regions are shown on the left side of each chromosome, and CNVs in macroscopically normal regions are shown on the right side of each chromosome. Red, gain in biparental-PMD specimens; orchid, gain in ABM-PMD specimens; blue, loss in biparental-PMD specimens; pale blue, loss in ABM-PMD specimens. None of the CNVs were obviously pathological and none included any imprinted DMRs analyzed in this study.

Fig. S5

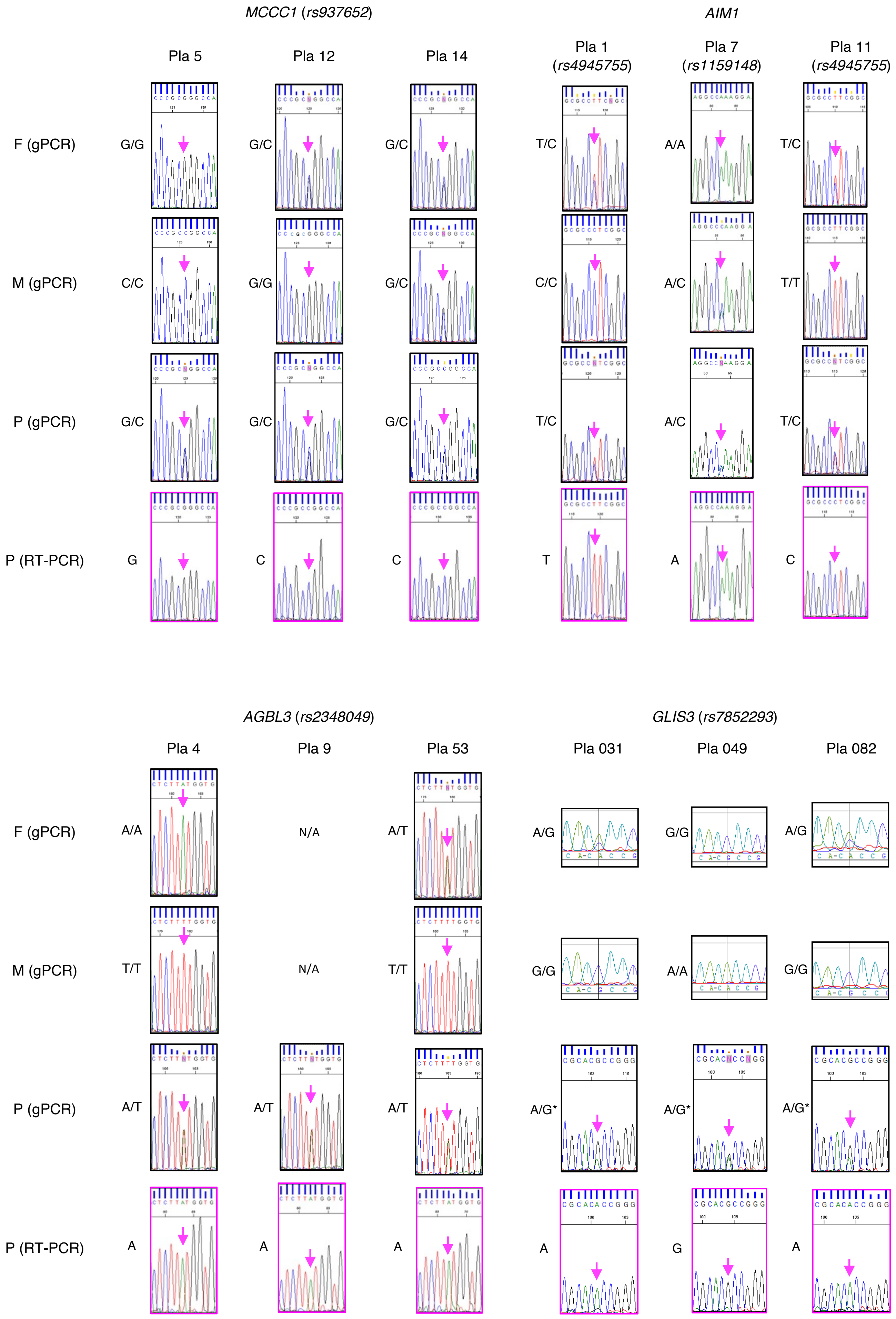
MCCC1 (rs937652)
AIM1
Pla 5
Pla 12
Pla 14
Pla 1 (rs4945755)
Pla 7 (rs1159148)
Pla 11 (rs4945755)
F (gPCR)
G/G
G/C
G/C
T/C
A/A
T/C
M (gPCR)
C/C
G/G
G/C
C/C
A/C
T/T
P (gPCR)
G/C
G/C
G/C
T/C
A/C
T/C
P (RT-PCR)
G
C
C
T
A
C
AGBL3 (rs2348049)
GLIS3 (rs7852293)
Pla 4
Pla 9
Pla 53
Pla 031
Pla 049
Pla 082
F (gPCR)
A/A
N/A
A/T
A/G
G/G
A/G
M (gPCR)
T/T
N/A
T/T
G/G
A/A
G/G
P (gPCR)
A/T
A/T
A/T
A/G*
A/G*
A/G*
P (RT-PCR)
A
A
A
A
G
A

Fig. S5 continued

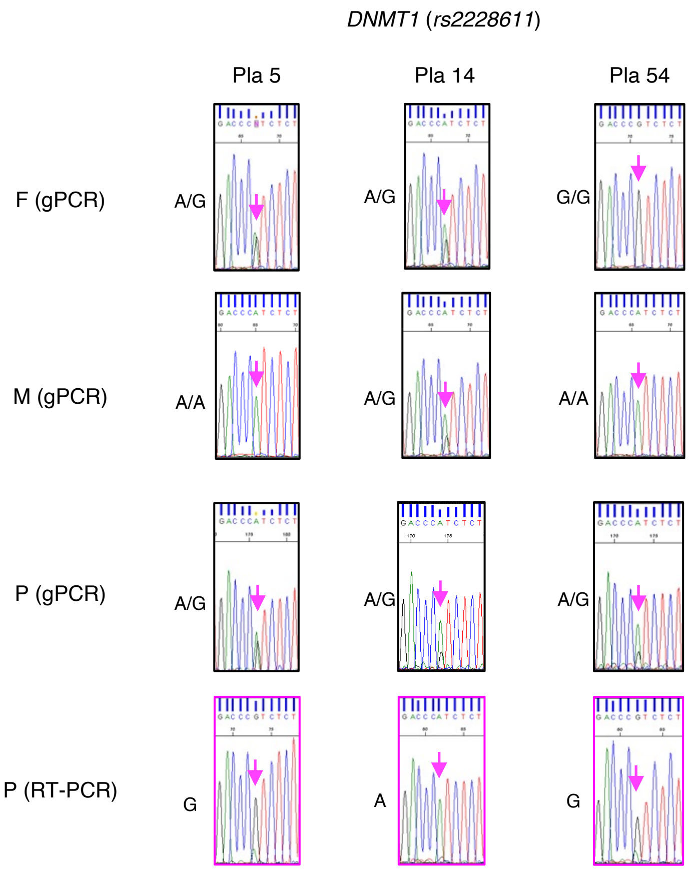

Supplementary Fig. S5. Preferential paternal expression of placenta-specific imprinted genes in normal placentas

Using single-nucleotide polymorphisms (SNPs), we screened the genotypes of the imprinted genes via polymerase chain reaction with genomic DNAs (gPCR) and examined their allelic expression via reverse-transcription PCR (RT-PCR). In the normal placentas, all genes except for *DNMT1* showed paternal expression. We observed very low expression of maternal alleles in *DNMT1* in Pla 5 and Pla 54. Arrows indicate the positions of the SNPs. F, father; M, mother; P, placenta; N/A, not available. *, genotype of cord blood.
